# Erythema Nodosum and Mycoplasma pneumoniae Infections in Childhood: Further Observations in Two Patients and a Literature Review

**DOI:** 10.14740/jocmr2011w

**Published:** 2015-02-09

**Authors:** Filippo Greco, Roberta Catania, Alice Le Pira, Marco Saporito, Luisa Scalora, Maria Giovanna Aguglia, Pierluigi Smilari, Giovanni Sorge

**Affiliations:** a Unit of Clinical Pediatrics, Department of Medical and Pediatric Sciences, University of Catania, Catania, Italy

**Keywords:** Erythema nodosum, *Mycoplasma pneumoniae*, Children

## Abstract

Erythema nodosum (EN) is the most frequent panniculitis in childhood and has been associated with various conditions, such as infectious and autoimmune disorders, medications, and malignancies. The author reports on two children affected with EN associated with *Mycoplasma pneumoniae* infection, which occurred in one patient without pulmonary detection. The available literature on EN and *M. pneumoniae* infection in childhood is also reviewed.

## Introduction

*Mycoplasma pneumoniae* is one of the most important bacterial agents of pneumonia in pediatric patients. It has been observed that *M. pneumoniae* can also affect other organs in the absence of respiratory localization. Among extrapulmonary manifestations, cutaneous involvement is more common and heterogeneous. We report two female patients with erythema nodosum (EN), 7 and 9 years of age, in whom serologic investigations revealed signs of active infection with *M. pneumoniae*. The available literature on EN and *M. pneumoniae* infection in childhood is reviewed.

## Case Reports

### Case 1

A 7-year-old female was referred for evaluation with a 10-day history of pain in the lower limbs, fevers, and multiple tender erythematous nodules on the legs. She was the firstborn of non-consanguineous parents; the family history was unremarkable and there was no consanguinity. She was born at 39 weeks gestation by vaginal delivery after an uncomplicated pregnancy. The birth weight was 2,950 g, the perinatal period was uneventful, and psychomotor development was normal.

Approximately 3 months before admission, she developed febrile tonsillitis that was treated with oral amoxicillin. At the time of admission to the Clinical Pediatric Division of the University of Catania, her weight was 60 kg (97th percentile), her height was 160 cm (75th percentile), and her head circumference was 56 cm (75th percentile). On clinical examination, she was alert and febrile. Multiple, round, tender erythematous nodules with a diameter of 2 cm and irregular indistinct borders were present bilaterally, mainly on the lower legs, but also on the arms. These findings were consistent with a diagnosis of EN. The cardiovascular and respiratory examinations were normal and the blood pressure was normal. The abdomen was non-tender and tractable; the liver and spleen were within normal size limits. On neurologic examination, there were no signs of meningeal irritation, and deep tendon reflexes were present and symmetric. Laboratory findings showed a normal leukocyte count (10,500/µL), a remarkable elevation of the erythrocyte sedimentation rate (ESR; 120 mm/h (normal < 12 mm/h)), and of C-reactive protein (CRP; 6.10 mg/dL (normal < 0.8 mg/dL)). Urinalysis showed microscopic persistent mild-to-moderate proteinuria (+ to ++ on dipstick) with normal renal function tests (urea, 4.5 mmol/L; creatinine, 47 μmol/L) and proteinuria values of 250 mg/dL per day. The following clinical investigations were all within the normal range: chest radiography, urinalysis, red blood cell count, platelet count, glucose, serum electrolytes, transaminases, bleeding time, fibrinogen, immunoglobulins, tuberculin skin test, antistreptolysin O (ASLO) titer, stool culture, antinuclear antibodies (ANA), and antibodies to *Salmonella typhi*, cytomegalovirus, Epstein-Barr virus (EBV), *Toxoplasma gondii*, and *Borrelia burgdorferi*. The titer of anti-*M. pneumoniae* antibodies, detected by a microparticle agglutination assay, was 1:160, and the serum immunoblot assay revealed positive IgM and IgG responses against *M. pneumoniae*. Therefore, specific therapy with oral clarithromycin for 10 days (15 mg/kg/day) was initiated, with progressive clinical improvement and complete recovery after 21 days from admission and gradual resolution of the proteinuria.

### Case 2

The patient was the second female child born to non-consanguineous parents. The family history was unremarkable. She was born at 37 weeks gestation by normal delivery after an uncomplicated pregnancy. The perinatal period was uneventful and psychomotor development was normal. At the age of 9 years 1 month, she developed fevers and a cough for 5 days that was treated with oral cephalosporin. After 15 days, two symmetric nodular lesions, which were painful on palpation and had a diameter of 3 cm, were observed on the tibial region. A topical steroid was administered without any improvement. After 1 month, because of the persistence of the cutaneous signs, the child was admitted to the Clinical Pediatric Division of the University of Catania. Her weight was 40 kg (90th percentile), her height was 135 cm (75th percentile), and her head circumference was 54 cm (50th percentile). On clinical examination, she was alert and afebrile. The clinical exam showed the presence of two painful erythematous nodules on the legs bilaterally. On respiratory examination, bibasilar rales were present, but the results were otherwise normal, including the cardiac, abdominal, and neurologic examinations. Chest X-ray results showed a bilateral interstitial infiltrate. The complete blood count showed 14,300 white cells/mm^3^ with 75% segmented neutrophils. The CRP and ESR were slightly increased, with values of 2.40 mg/dL (normal < 0.8 mg/dL) and 45 mm/h (normal < 12 mm/h). Normal values were recorded for hematocrit, platelet count, and glucose, sodium, potassium, creatinine, plasma urea, creatine kinase, pancreatic, and liver enzyme levels. The following clinical investigations were all within the normal range: coagulation profile; immunoglobulins; tuberculin skin test; ASLO titer; and antibodies to *S. typhi*, cytomegalovirus, EBV, *Chlamydia pneumoniae*, and *B. burgdorferi*. The serum titer of antibodies to *M. pneumoniae*, as assessed by a microparticle agglutination assay, was 1:320. Serum immunoblot assays revealed positive IgM (1:120) and IgG (1:520) against *M. pneumoniae*. A diagnosis of pulmonary *M. pneumoniae* infection with EN was made and specific treatment with oral clarithromycin, at a dosage of 15 mg/kg/day for 2 weeks was initiated. Within 7 days, the cutaneous and respiratory neurologic signs had improved and the patient was discharged after 10 days with a full clinical recovery.

## Discussion

*M. pneumoniae* is a common intracellular pathogen which is responsible for respiratory tract diseases. *M. pneumoniae* can also give rise to other manifestations, with or without pulmonary involvement, which is the result of direct invasion and/or an autoimmune response [1]. The major extrapulmonary manifestations of *M. pneumoniae* infections described in the literature are neurologic (encephalitis, myelitis, Guillain-Barre syndrome, stroke, and acute disseminated encephalomyelitis), cardiovascular (pericarditis, myocarditis, and endocarditis), hematologic (autoimmune hemolytic anemia, aplastic anemia, thrombocytopenic purpura, and hemophagocytic syndrome), gastrointestinal (hepatitis, pancreatitis, and gastroenteritis), musculoskeletal (arthritis and rhabdomyolysis), renal (glomerulonephritis and severe proteinuria) and dermatologic [2-6]. Cutaneous manifestations occur in 10-25% of all *M. pneumoniae* infections and include non-specific exanthems, urticaria, vasculitis, Stevens-Johnson syndrome, toxic epidermal necrolysis, pityriasis rosea, and EN [7]. EN is the most common type of panniculitis in childhood and is defined as a hypodermal septal inflammation which causes the appearance of erythematous, tender, subcutaneous nodules. The classic location of the subcutaneous nodules is the bilateral tibial region; involvement of the thighs, face, upper limbs, and trunk has also been reported in children [8]. It is considered a self-limited autoimmune disease associated with many disorders, including infections, such as streptococcal pharyngitis, EBV, cytomegalovirus, and tuberculosis, rheumatologic diseases, inflammatory bowel disease, medications, autoimmune disorders, and malignancies. The first study which focused on the etiology of EN in childhood was a retrospective study reported by Labbe et al [9] involving 27 pediatric patients with EN; in nearly one-half of the patients, the cause of EN remained unclear, while streptococcal infection represented the most frequent etiologic agent, followed by yersiniosis, salmonellosis, and sarcoidosis. Recently, among the various etiological agents related to EN reported in the literature have mostly been adult patients with documented infection by *M. pneumoniae*, both with and without pulmonary involvement. In the pediatric age group, the first description of *M. pneumoniae* infection and EN was in 2001 by Kakourou et al [10], which is a case series involving 35 children (age range, 1.3 - 14 years; mean age, 8.9 ± 3.4 years) with EN; three children (7, 9, and 13 years of age) had antibodies against mycoplasma indicative of recent infection [10]. Of these patients, only one had signs of respiratory involvement, while in the second patient there was an associated fever, and no other symptoms were present in the third patient.

Blanco et al [11] described the case of a 4-year-old child approximately 15 days after the onset of a pulmonary infectious process who presented with cutaneous manifestations in the region and on the back of the tibia, compatible with a foot EN. Shimuzu et al [12] reported an 8-year-old girl, who 7 days after the onset of EN had a diffuse maculopapular rash like erythema multiforme, followed by Schonlein-Henoch purpura, all in the absence of pulmonary manifestations (clinically and radiologically). In the last study performed by Aydin-Teke et al [13], a total of 39 EN patients were retrospectively evaluated; streptococcal infections were the most common cause, followed by tularemia, tuberculosis, and miscellaneous conditions (Behcet disease, cytomegalovirus, *Giardia lamblia* infection, and sarcoidosis). Two of 39 patients were infected with *M. pneumoniae*, and one had a *M. pneumoniae* infection plus a concomitant acute streptococcal infection.

In both of the cases reported herein, *M. pneumoniae* infection was diagnosed on the basis of immunoglobulin M-positive serology. In the first patient, there was simultaneous renal impairment with mild proteinuria, a feature which is quite rare in the literature and usually present during pneumonia. In the second patient, cutaneous manifestations were observed with pulmonary involvement. The majority of cases reported in the literature, including the two cases reported herein, were treated with clarithromycin and had good clinical outcomes, and none had a relapse of symptoms. Table 1 describes the main clinical findings of children with EN associated with *M. pneumoniae* infection, as reported in the literature. To our knowledge, this is the first review of the literature on EN and *M. pneumoniae* infection in children. The pathogenesis of EN is not fully understood: it has been speculated that *M. pneumoniae* can be transferred hematogenously to the dermis, thus causing hypodermal inflammation. Nevertheless, it is not possible to exclude an immunologic mechanism, such as autoimmunity [13]. In conclusion, we recommend that *M. pneumoniae* should be included routinely in screening of children with cutaneous manifestations, and in particular children with EN, even in the absence of respiratory involvement. Further clinical and immunologic studies are necessary to better understand the pathogenic mechanism of extrapulmonary manifestations of *M. pneumoniae* infections.

**Table 1:** Main Clinical Findings of Children With Erythema Nodosum Associated With *Mycoplasma pneumoniae* Infection Reported in the Literature

References	Age at observation	Clinical findings/localization	Others findings	Treatment	Outcome
Kakorurou et al, 2001 [10]	9 years 13 years 7 years	Fever/legs and arms Fever/legs Legs and thighs	- Cough, arthralgias -		Good
Blanco et al, 2002 [11]	4 years	Fever/bilateral lower legs and foot	Pneumonia	Clarithromycin	Good
Shimuzu et al, 2012 [12]	8 years	Bilateral lower legs	Arthralgias, erythema multiforme, Henoch-Schonlein purpura	Clarithromycin	Good
Aydin-Teke et al, 2014 [13]	10 years 6 years 8 years	Bilateral lower legs Bilateral lower legs Bilateral lower legs	Concomitant streptococcal infection NR NR	NR NR NR	NR NR NR
Present study, patient 1	7 years	Fever/bilateral legs	Proteinuria	Clarithromycin	Good
Present study, patient 2	9 years	Bilateral legs and arms	Pneumonia	Clarithromycin	Good

NR: not reported.
